# Cell cycle progression score is a marker for five-year lung cancer-specific mortality risk in patients with resected stage I lung adenocarcinoma

**DOI:** 10.18632/oncotarget.9129

**Published:** 2016-05-02

**Authors:** Takashi Eguchi, Kyuichi Kadota, Jamie Chaft, Brent Evans, John Kidd, Kay See Tan, Joe Dycoco, Kathryn Kolquist, Thaylon Davis, Stephanie A. Hamilton, Kraig Yager, Joshua T. Jones, William D. Travis, David R. Jones, Anne-Renee Hartman, Prasad S. Adusumilli

**Affiliations:** ^1^ Thoracic Service, Department of Surgery, Memorial Sloan Kettering Cancer Center, New York, NY, USA; ^2^ Division of Thoracic Surgery, Department of Surgery, Shinshu University School of Medicine, Matsumoto, Japan; ^3^ Department of Pathology, Memorial Sloan Kettering Cancer Center, New York, NY, USA; ^4^ Department of Diagnostic Pathology, Faculty of Medicine, Kagawa University, Kagawa, Japan; ^5^ Department of Medicine, Memorial Sloan Kettering Cancer Center, New York, NY, USA; ^6^ Myriad Genetic Laboratories, Inc., Salt Lake City, UT, USA; ^7^ Department of Epidemiology and Biostatistics, Memorial Sloan Kettering Cancer Center, New York, NY, USA; ^8^ Center for Cell Engineering, Memorial Sloan Kettering Cancer Center, New York, NY, USA

**Keywords:** molecular prognostic score, CCP score, chemotherapy, adjuvant therapy, overall survival

## Abstract

**Purpose:**

The goals of our study were (a) to validate a molecular expression signature (cell cycle progression [CCP] score and molecular prognostic score [mPS; combination of CCP and pathological stage {IA or IB}]) that identifies stage I lung adenocarcinoma (ADC) patients with a higher risk of cancer-specific death following curative-intent surgical resection, and (b) to determine whether mPS stratifies prognosis within stage I lung ADC histological subtypes.

**Methods:**

Formalin-fixed, paraffin-embedded stage I lung ADC tumor samples from 1200 patients were analyzed for 31 proliferation genes by quantitative RT-PCR. Prognostic discrimination of CCP score and mPS was assessed by Cox proportional hazards regression, using 5-year lung cancer–specific mortality as the primary outcome.

**Results:**

In multivariable analysis, CCP score was a prognostic marker for 5-year lung cancer–specific mortality (HR=1.6 per interquartile range; 95% CI, 1.14–2.24; *P*=0.006). In a multivariable model that included mPS instead of CCP, mPS was a significant prognostic marker for 5-year lung cancer–specific mortality (HR=1.77; 95% CI, 1.18–2.66; *P*=0.006). Five-year lung cancer–specific survival differed between low-risk and high-risk mPS groups (96% vs 81%; *P*<0.001). In patients with intermediate-grade lung ADC of acinar and papillary subtypes, high mPS was associated with worse 5-year lung cancer–specific survival (*P*<0.001 and 0.015, respectively), compared with low mPS.

**Conclusion:**

This study validates CCP score and mPS as independent prognostic markers for lung cancer–specific mortality and provides quantitative risk assessment, independent of known high-risk features, for stage I lung ADC patients treated with surgery alone.

## INTRODUCTION

The estimated 5-year overall survival (OS) for patients with stage IA and IB lung adenocarcinoma (ADC) is 81%–87% and 72%, respectively [1], despite curative-intent surgical resection. Whether adjuvant therapy would improve OS among patients with stage I lung ADC is undetermined. Current National Comprehensive Cancer Network (NCCN) guidelines recommend adjuvant therapy for patients with stage IB lung ADC on the basis of tumor size; this follows results from the CALGB 9633 trial [2]. Recent findings from our group and others indicate that size alone may not be adequate when determining tumor aggressiveness [3–8].

To expand prognostic information beyond tumor size alone, the International Association for the Study of Lung Cancer, American Thoracic Society, and European Respiratory Society (IASLC/ATS/ERS) proposed a new lung ADC classification in 2011 [9]. which has been validated in independent cohorts [3, 10, 11] and is now adopted by the 2015 WHO classification [12]. A major limitation of this classification is that the majority (40%–70%) of cases of stage I lung ADC are intermediate-grade acinar predominant (ACI) or papillary predominant (PAP) subtypes [11, 13, 14]. An objective, quantitative, reproducible molecular expression signature that is applicable to both stage IA and IB lung ADC tumors and that can be used to further stratify intermediate-grade ADCs, to better identify high-risk patients, would be beneficial.

A 46-gene panel was developed to assess cell cycle gene expression, and generate a cell cycle progression (CCP) score for pathological stage I and II lung ADC tumors [15–18]. CCP score was previously shown to be a prognostic factor for lung ADC mortality in combined cohorts of patients with stage I or II lung ADC [16–18]. Using a cohort of 650 patients with stage I or II disease, a molecular prognostic score (mPS; inclusive of CCP score and stage) was developed to provide quantitative prognostic information [17].

In our study, we assessed the ability of CCP score and mPS to predict lung cancer–specific mortality in a cohort of 1200 patients with stage I ADC treated with surgery alone. The use of this well-annotated, previously reported cohort allowed us to assess the prognostic ability of all known clinical, surgical, pathological, histological, and molecular features indicative of aggressiveness—which included lymphatic and vascular invasion [4], type of surgical procedure [7, 19], histological subtypes (defined in the IASLC/ATS/ERS classification) [3, 7, 19], and *EGFR* and *KRAS* mutation status [20]. This expansive analysis, inclusive of all known high-risk factors, demonstrated that CCP score and mPS are independent prognostic markers for patients with stage I lung ADC.

## RESULTS

### Evaluable analysis set

**Figure 1:**
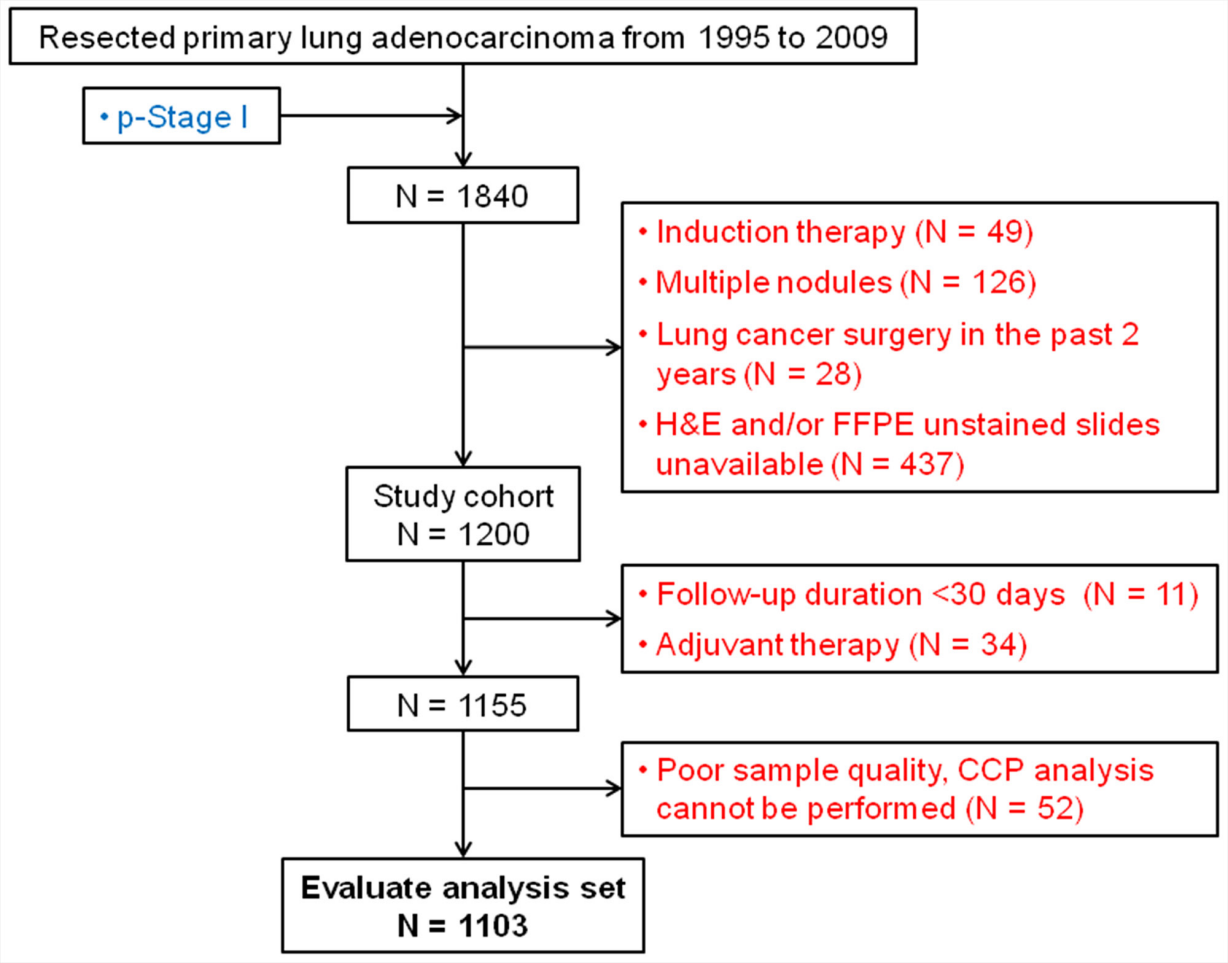
Disposition schematic The evaluable analysis set included all patients who have a follow-up duration ≥30 days, did not receive adjuvant therapy, have a cell cycle progression (CCP) score, and have follow-up information. FFPE, formalin-fixed, paraffin-embedded; H&E, hematoxylin and eosin.

Among the 1200 patients with samples, 11 (0.9%) had follow-up <30 days after surgery, 34 (2.8%) received adjuvant therapy, and 52 (4.3%) had tumor samples for which a CCP score could not be computed. Among the evaluable analysis set (Figure 1; N = 1103), patients had a median age at diagnosis of 69 years (Table 1). The majority of patients were women (61.0%), former smokers (68.1%), and had stage IA disease (72.4%). Since the number of bilobectomies and pneumonectomies was small (n < 10), these two surgical procedures were combined with lobectomies when the surgical procedure variable was defined.

**Table 1:** Patient clinical characteristics

Characteristic	Evaluable analysis set (N = 1103)
Age at diagnosis (years)	
Mean (SD)	68.3 (10.0)
Median	69
Min, Max	23, 96
Sex	
Male	430 (39.0)
Female	673 (61.0)
Smoking status	
Never	192 (17.4)
Former	751 (68.1)
Current	160 (14.5)
Surgical procedure	
Pneumonectomy/bilobectomy/lobectomy	824 (74.7)
Segmentectomy	96 (8.7)
Wedge resection	183 (16.6)
Tumor size (centimeters)	
Mean (SD)	2.1 (1.0)
Median	2.0
Min, Max	0.3, 5.0
Pathological stage	
IA	799 (72.4)
IB	304 (27.6)
Pleural invasion	
PLX/PL0	931 (84.4)
PL1	154 (14.0)
PL2	18 (1.6)
Lymphatic invasion	
Absent	750 (68.0)
Present	353 (32.0)
Vascular invasion	
Absent	812 (73.6)
Present	291 (26.4)
Morphological grade	
Low	156 (14.1)
Intermediate	690 (62.6)
High	257 (23.3)

Note. Data are no. (%), unless otherwise noted. Max = maximum, Min = minimum, SD = standard deviation.

### CCP score and 5-year lung cancer–specific mortality

On univariable analysis, CCP score (*P* < 0.001) and all clinical variables—except sex and smoking status—were significant prognostic factors for 5-year lung cancer–specific mortality (Table 2). On multivariable analysis, CCP score was an independent significant prognostic marker (*P* = 0.006) for 5-year lung cancer–specific mortality (hazard ratio [HR] = 1.6; 95% CI = 1.14–2.24; Table 3). Other significant variables in the multivariable analysis were age at diagnosis (*P* = 0.016), surgical procedure (*P* < 0.001), tumor size (*P* < 0.001), lymphatic invasion (*P* = 0.02), and morphological grade (*P* = 0.012).

**Table 2:** Univariable Cox proportional hazards regression analyses of cell cycle progression (CCP) score, molecular prognostic score (mPS), and clinical characteristics with 5-year lung cancer mortality in the evaluable analysis set

Variable	Hazard ratio (95% CI)	*P* value
CCP score	2.46 (1.85, 3.29)	<0.001
mPS	2.67 (2.08, 3.42)	<0.001
Age at diagnosis	1.03 (1.01, 1.06)	0.003
Sex		0.065
Male	1	
Female	0.68 (0.45, 1.03)	
Smoking status		0.109
Never	1	
Former	1.94 (1.02, 4.17)	
Current	2.06 (0.92, 4.91)	
Surgical procedure		<0.001
Pneumonectomy/bilobectomy/lobectomy	1	
Segmentectomy	2.11 (1.07, 3.81)	
Wedge resection	2.79 (1.74, 4.38)	
Tumor size	1.56 (1.29, 1.87)	<0.001
Pathological stage		<0.001
IA	1	
IB	3.65 (2.43, 5.53)	
Pleural invasion		<0.001
PLX/PL0	1	
PL1	3.29 (2.06, 5.14)	
PL2	6.12 (2.36, 13.05)	
Lymphatic invasion		<0.001
Absent	1	
Present	3.32 (2.20, 5.06)	
Vascular invasion		<0.001
Absent	1	
Present	3.16 (2.09, 4.76)	
Morphological grade		<0.001
Low	1	
Intermediate	5.74 (1.78, 35.11)	
High	13.41 (4.13, 82.37)	

Note. Hazard ratios for CCP score and mPS are per the interquartile range of each score variable, respectively.

CI = confidence interval.

**Table 3:** Multivariable Cox proportional hazards regression analysis of cell cycle progression (CCP) score, molecular prognostic score (mPS), and clinical characteristics with 5-year lung cancer mortality in the evaluable analysis set

Variable	Analysis with CCP score		Analysis with mPS	
	Hazard ratio (95% CI)	*P* value	Hazard ratio (95% CI)	*P* value
CCP score	1.60 (1.14, 2.24)	0.006		
mPS			1.77 (1.18, 2.66)	0.006
Age at diagnosis	1.03 (1.01, 1.05)	0.016	1.03 (1.01, 1.05)	0.016
Sex		0.343		0.342
Male	1		1	
Female	0.82 (0.54, 1.25)		0.82 (0.54, 1.24)	
Smoking status		0.676		0.675
Never	1		1	
Former	1.35 (0.69, 2.95)		1.35 (0.69, 2.95)	
Current	1.41 (0.60, 3.51)		1.41 (0.60, 3.51)	
Surgical procedure		<0.001		<0.001
Pneumonectomy/Bilobectomy/Lobectomy	1		1	
Segmentectomy	3.31 (1.62, 6.30)		3.31 (1.62, 6.30)	
Wedge resection	4.65 (2.71, 7.91)		4.65 (2.71, 7.90)	
Tumor size	1.64 (1.24, 2.14)	<0.001	1.64 (1.24, 2.15)	<0.001
Pathological stage		0.720		0.158
IA	1		1	
IB	0.87 (0.39, 1.90)		0.53 (0.22, 1.28)	
Pleural invasion		0.056		0.057
PLX/PL0	1		1	
PL1	1.94 (0.91, 4.12)		1.94 (0.91, 4.12)	
PL2	3.39 (1.15, 8.79)		3.38 (1.15, 8.76)	
Lymphatic invasion		0.020		0.019
Absent	1		1	
Present	1.73 (1.09, 2.77)		1.74 (1.10, 2.78)	
Vascular invasion		0.089		0.091
Absent	1		1	
Present	1.52 (0.94, 2.44)		1.51 (0.94, 2.44)	
Morphological grade		0.012		0.011
Low	1		1	
Intermediate	3.08 (0.92, 19.13)		3.08 (0.92, 19.13)	
High	5.09 (1.46, 32.18)		5.10 (1.46, 32.22)	

Note. Hazard ratios for CCP score and mPS are per the interquartile range of each score variable, respectively.

CI = confidence interval.

### mPS and 5-year lung cancer–specific mortality

On univariable analysis, mPS (*P* < 0.001) was a significant prognostic factor for 5-year lung cancer–specific mortality (Table 2). On multivariable analysis, mPS was an independent significant prognostic marker (*P* = 0.006) for 5-year lung cancer–specific mortality (HR = 1.77; 95% CI = 1.18–2.66; Table 3). Other significant variables included age at diagnosis (*P* = 0.016), surgical procedure (*P* < 0.001), tumor size (*P* < 0.001), lymphatic invasion (*P* = 0.019), and histological grade (*P* = 0.011).

**Figure 2:**
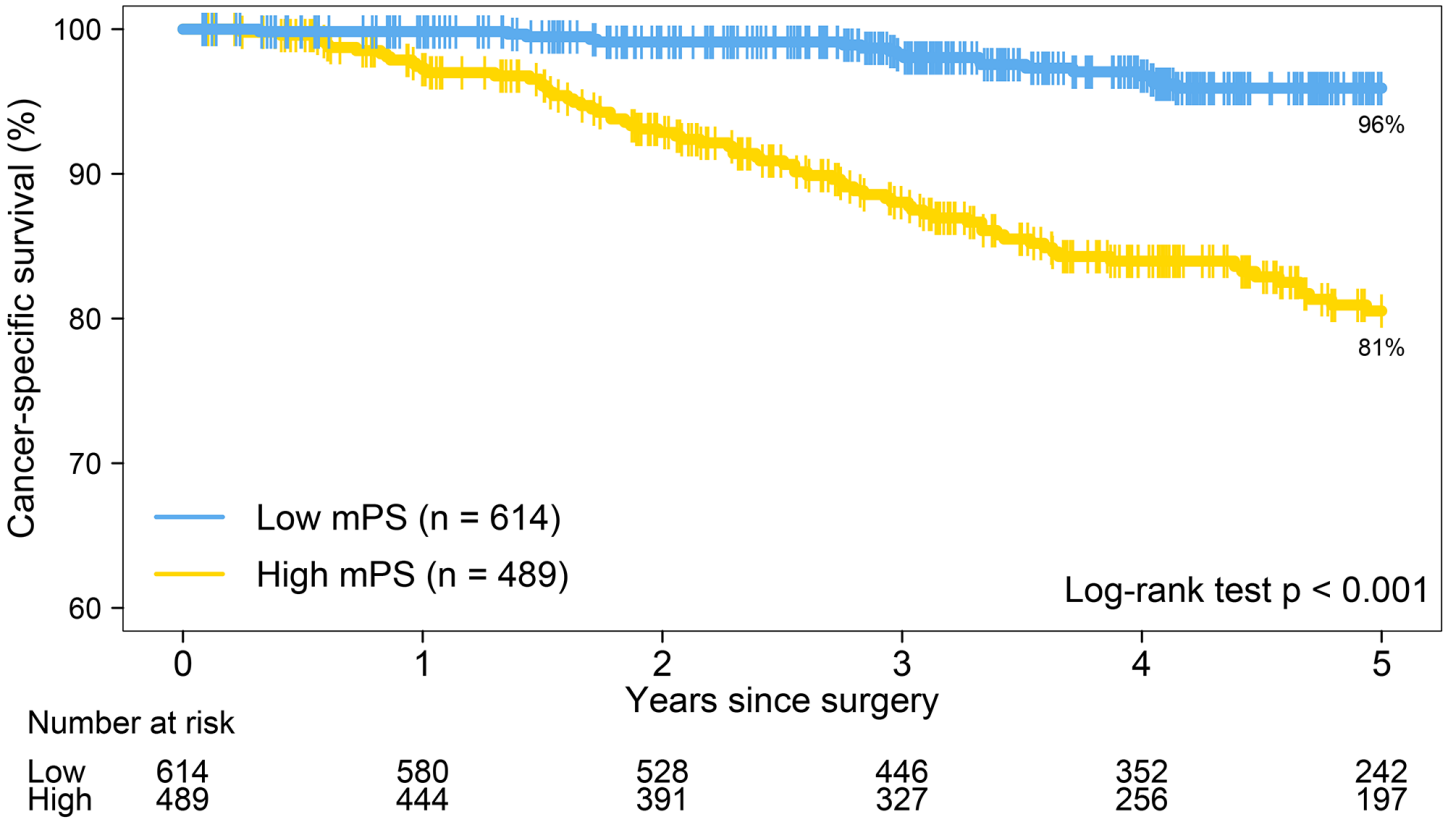
The Kaplan-Meier survival estimates for patients with low molecular prognostic score (mPS; N = 614) and high mPS (N = 489) show that the 5-year lung cancer–specific survival rate is 96% for patients with low mPS and 81% for patients with high mPS (*P* < 0.001)

When a predefined threshold of 27 was used to define the high/low mPS variable [17, 18], the evaluable analysis set had 614 low-mPS patients and 489 high-mPS patients. The difference in 5-year lung cancer–specific survival was statistically significant (*P* < 0.001): 96% for low-mPS patients vs 81% for high-mPS patients (Figure 2).

### mPS and histological subtypes

**Figure 3:**
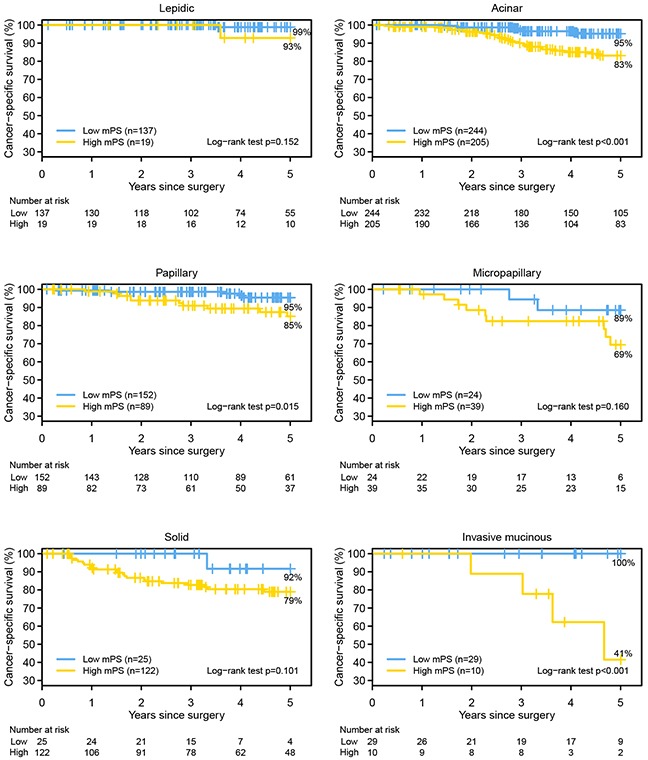
The Kaplan-Meier survival estimates for the high and low molecular prognostic score (mPS) groups, by morphological subtype, are shown The 5-year lung cancer–specific survival for low mPS vs high mPS for each subtype: lepidic predominant, 99% vs 93% (*P* = 0.152); acinar predominant, 95% vs 83% (*P* < 0.001); papillary predominant, 95% vs 85% (*P* = 0.015); micropapillary predominant, 89% vs 69% (*P* = 0.16); solid predominant, 92% vs 79% (*P* = 0.101); and invasive mucinous adenocarcinoma, 100% vs 42% (*P* < 0.001).

Figure 3 shows the 5-year lung cancer–specific survival curves for the high- and low-mPS groups by predominant histological subtype. For all cases, patients with high mPS had worse lung cancer–specific survival than those with low mPS; however, only ACI (*P* < 0.001), PAP (*P* = 0.015), and invasive mucinous adenocarcinoma (IMA) (*P* < 0.001) tumors had statistically significant differences between high and low mPS.

**Figure 4:**
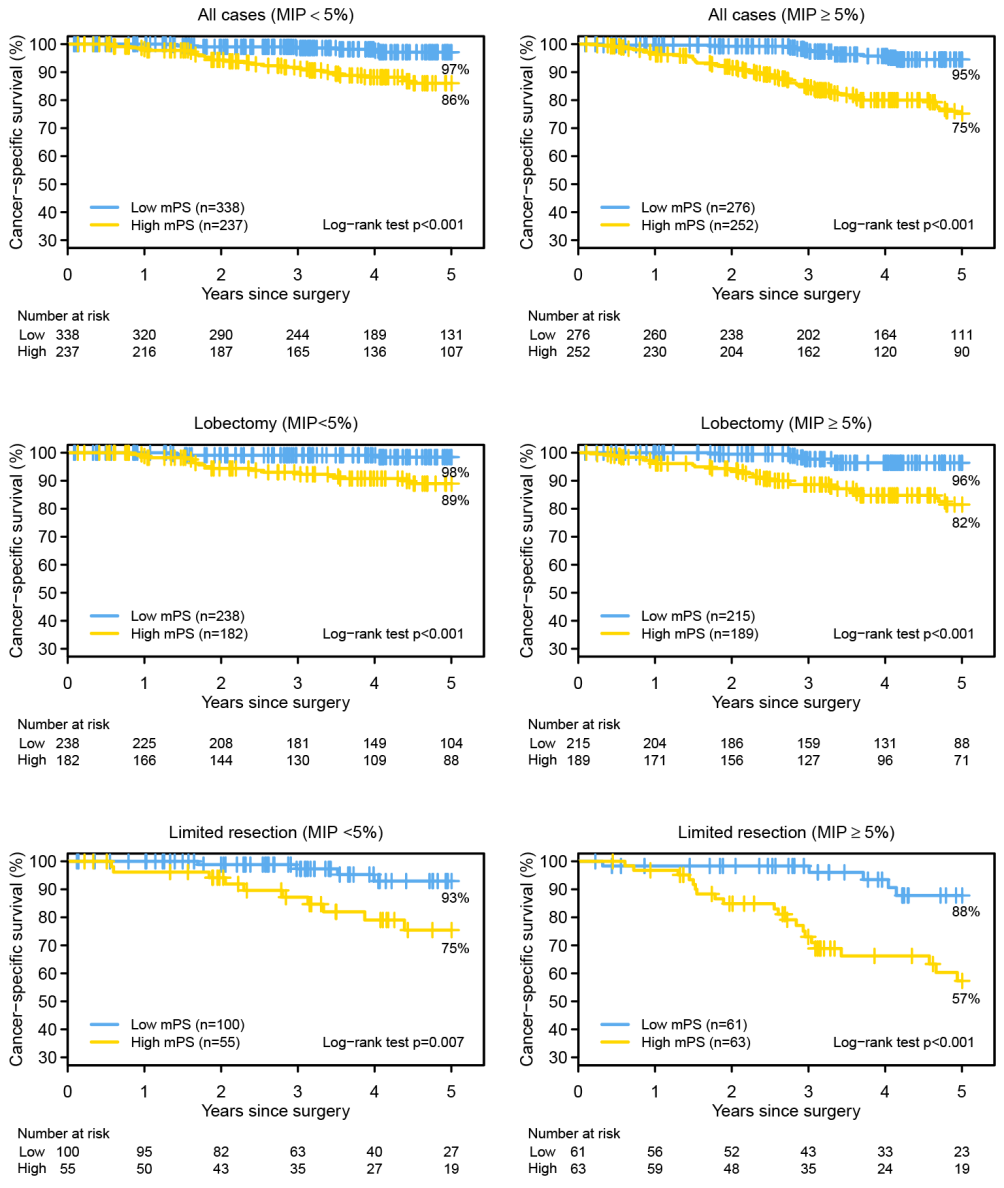
The Kaplan-Meier survival estimates for the high and low molecular prognostic score (mPS) groups, by the absence (<5%) or presence (≥5%) of micropapillary (MIP) pattern and surgical procedure, are shown The 5-year lung cancer–specific survival for low mPS vs high mPS: all cases (MIP < 5%), 97% vs 86% (*P* < 0.001); all cases (MIP ≥ 5%), 95% vs 75% (*P* < 0.001); lobectomy (MIP < 5%), 98% vs 89% (*P* < 0.001); lobectomy (MIP ≥ 5%), 96% vs 82% (*P* < 0.001); limited resection (MIP < 5%), 93% vs 75% (*P* = 0.007); and limited resection (MIP ≥ 5%), 88% vs 57% (*P* < 0.001).

On the basis of our and others' previous publications that demonstrated worse prognosis in patients with micropapillary (MIP) subtype tumors who had undergone limited resection compared with those who had undergone lobectomy [19, 21], we investigated the utility of mPS in this cohort of patients (Figure 4). Within each setting, patients with high mPS had significantly lower survival estimates (*P* < 0.001). This was particularly true for patients with presence of the MIP subtype (≥5%)—5-year lung cancer–specific survival estimates were 95% for the low-mPS group vs 75% for the high-mPS group (*P* < 0.001). This difference was particularly pronounced among patients who had undergone limited resection (MIP ≥ 5%); the 5-year lung cancer–specific survival estimate was 88% for the low-mPS group vs 57% for the high-mPS group (*P* < 0.001).

**Figure 5:**
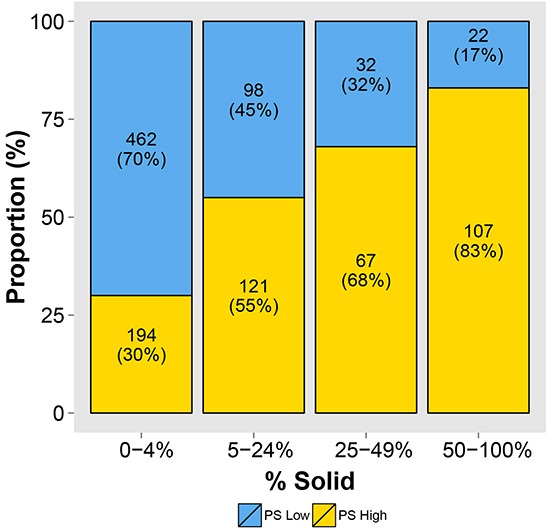
The relationship between high/low molecular prognostic score (mPS) and categories of increasing solid (SOL) pattern shows that, as the SOL pattern of the tumor increases, the proportion of high mPS increases (*P* < 0.001)

The relationship between high mPS/low mPS and cohorts of patients with increasing percentage of solid (SOL) pattern in their tumors is shown in Figure 5. As the percentage of SOL pattern in the tumor increases, the proportion of patients with high mPS increases (*P* < 0.001).

### Relationship between mPS and mutation status

**Figure 6:**
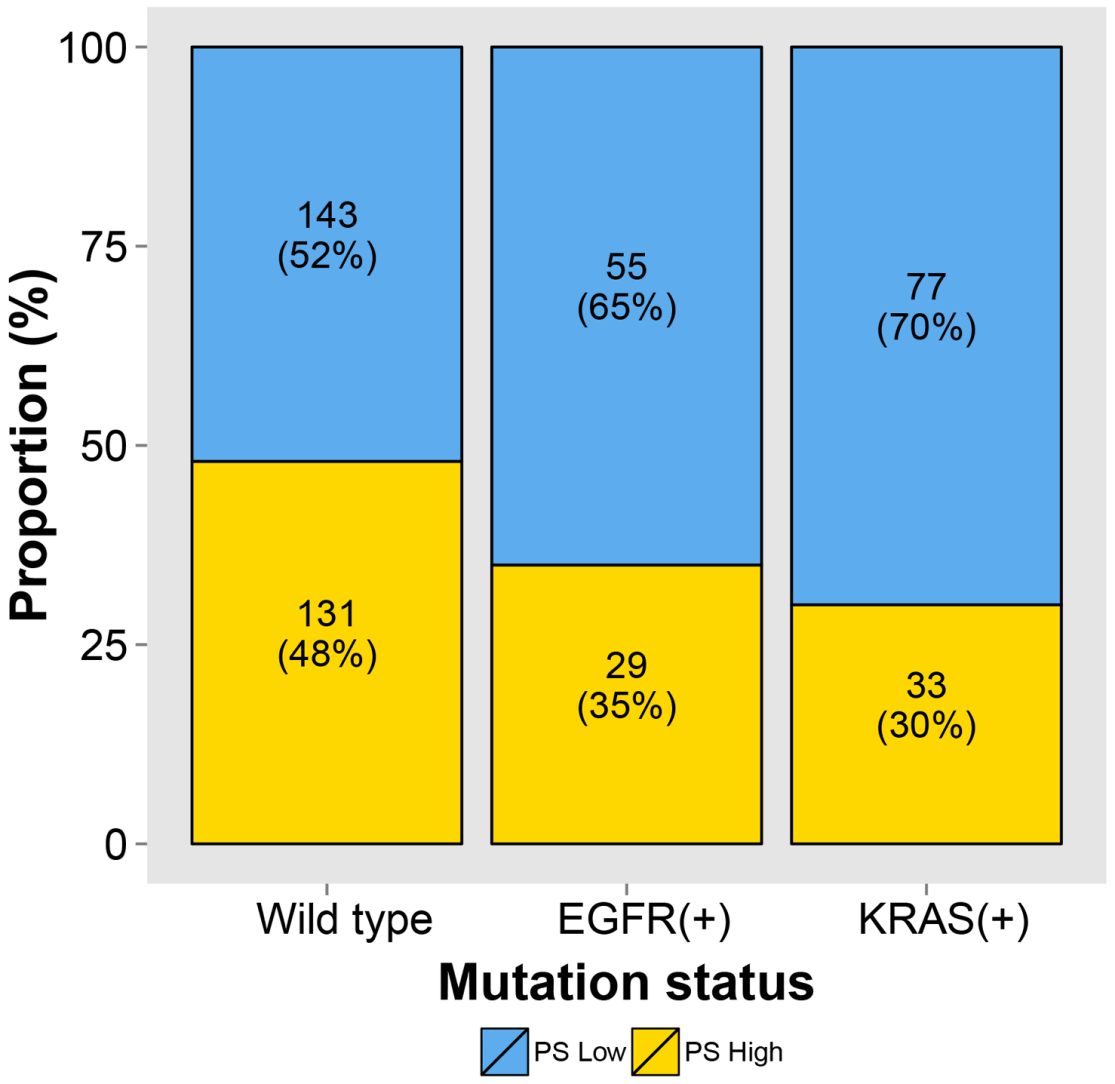
The relationship between high/low molecular prognostic score (mPS) and mutation status shows that wild-type tumors had an approximately equal proportion of high versus low mPS (48% vs 52%), whereas high mPS was less common in tumors with *EGFR* (35%) or *KRAS* (30%) mutations (*P* = 0.002)

Figure 6 shows the relationship between high mPS/low mPS and mutation status. Wild-type tumors had approximately equal proportions of high mPS and low mPS (48% vs 52%); however, high mPS was less common in tumors with *EGFR* (35%) or *KRAS* (30%) mutations (*P* = 0.002).

### Exploratory analyses with OS

Exploratory analyses were performed to evaluate CCP score and mPS as prognostic markers for death from any cause in patients with stage I lung ADC, after adjustment for clinical variables, including pathological stage. Univariable analyses showed that CCP score, mPS, and all evaluated clinical variables—except smoking status—were significant prognostic factors for 5-year OS (Table 4). On multivariable analysis, CCP score was an independent significant prognostic marker for 5-year OS (HR = 1.33; 95% CI = 1.06–1.67; *P* = 0.014). Other significant variables included age at diagnosis (*P* < 0.001), sex (*P* = 0.019), surgical procedure (*P* < 0.001), tumor size (*P* < 0.001), and pleural invasion (*P* = 0.017) (Table 5). In a separate multivariable analysis, mPS was an independent significant prognostic marker for 5-year OS (HR = 1.41; 95% CI = 1.07–1.85; *P* = 0.016).

**Table 4:** Univariable Cox proportional hazards regression analyses of cell cycle progression (CCP) score, molecular prognostic score (mPS), and clinical characteristics with overall survival in the evaluable analysis set

Variable	Hazard ratio (95% CI)	*P* value
CCP score	1.73 (1.41, 2.11)	<0.001
mPS	1.88 (1.58, 2.23)	<0.001
Age at diagnosis	1.05 (1.03, 1.07)	<0.001
Sex		<0.001
Male	1	
Female	0.61 (0.46, 0.81)	
Smoking status		0.190
Never	1	
Former	1.44 (0.96, 2.26)	
Current	1.48 (0.87, 2.53)	
Surgical procedure		<0.001
Pneumonectomy/bilobectomy/lobectomy	1	
Segmentectomy	1.62 (1.00, 2.49)	
Wedge resection	2.08 (1.48, 2.86)	
Tumor size	1.37 (1.19, 1.56)	<0.001
Pathological stage		<0.001
IA	1	
IB	2.44 (1.83, 3.23)	
Pleural invasion		<0.001
PLX/PL0	1	
PL1	2.71 (1.95, 3.70)	
PL2	3.06 (1.30, 6.07)	
Lymphatic invasion		<0.001
Absent	1	
Present	2.15 (1.62, 2.84)	
Vascular invasion		<0.001
Absent	1	
Present	2.31 (1.74, 3.06)	
Morphological grade		<0.001
Low	1	
Intermediate	1.95 (1.16, 3.55)	
High	3.01 (1.74, 5.59)	

Note. Hazard ratios for CCP score and mPS are per the interquartile range of each score variable, respectively.

CI = confidence interval.

**Table 5:** Multivariable Cox proportional hazards regression analysis of cell cycle progression (CCP) score, molecular prognostic score (mPS), and clinical characteristics with overall survival in the evaluable analysis set

Variable	Analysis with CCP score		Analysis with mPS	
	Hazard ratio (95% CI)	*P* value	Hazard ratio (95% CI)	*P* value
CCP score	1.33 (1.06, 1.67)	0.014		
mPS			1.41 (1.07, 1.85)	0.016
Age at diagnosis	1.04 (1.03, 1.06)	<0.001	1.04 (1.03, 1.06)	<0.001
Sex		0.019		0.019
Male	1		1	
Female	0.71 (0.53, 0.95)		0.71 (0.53, 0.95)	
Smoking status		0.468		0.464
Never	1		1	
Former	1.18 (0.78, 1.87)		1.18 (0.78, 1.87)	
Current	1.42 (0.81, 2.50)		1.42 (0.81, 2.50)	
Surgical procedure		<0.001		<0.001
Pneumonectomy/Bilobectomy/Lobectomy	1		1	
Segmentectomy	2.03 (1.24, 3.20)		2.03 (1.24, 3.19)	
Wedge resection	2.45 (1.69, 3.52)		2.45 (1.69, 3.51)	
Tumor size	1.42 (1.17, 1.72)	<0.001	1.42 (1.17, 1.72)	<0.001
Pathological stage		0.331		0.073
IA	1		1	
IB	0.76 (0.43, 1.32)		0.56 (0.30, 1.05)	
Pleural invasion		0.017		0.017
PLX/PL0	1		1	
PL1	2.13 (1.23, 3.70)		2.13 (1.23, 3.70)	
PL2	2.35 (0.92, 5.30)		2.35 (0.92, 5.29)	
Lymphatic invasion		0.091		0.089
Absent	1		1	
Present	1.32 (0.96, 1.82)		1.32 (0.96, 1.83)	
Vascular invasion		0.074		0.073
Absent	1		1	
Present	1.36 (0.97, 1.90)		1.36 (0.97, 1.90)	
Morphological grade		0.216		0.209
Low	1		1	
Intermediate	1.32 (0.77, 2.45)		1.33 (0.77, 2.46)	
High	1.65 (0.91, 3.20)		1.66 (0.91, 3.22)	

Note. Hazard ratios for CCP score and mPS are per the interquartile range of each score variable, respectively.

CI = confidence interval.

## DISCUSSION

Our study validates both CCP score and mPS as independent prognostic markers for lung cancer–specific mortality and OS in a large, uniform cohort of patients with stage I lung ADC treated with surgery alone. Since CCP score is a quantitative prognostic measure and derivative of multiple cell cycle genes, it may provide a more reliable and objective measure for lung cancer–specific mortality than the currently reported subjective variables, such as lymphatic and vascular invasion and histological grade. Our selection of 5-year lung cancer–specific mortality as the primary endpoint is relevant for the design of prospective studies investigating the potential benefit of adjuvant therapy for these patients.

Our study is distinct from previously published studies detailing the utility of CCP score and mPS, for the following reasons: (1) this is the first study to analyze a prognostic molecular signature in a large, uniform cohort of patients with stage I lung ADC; (2) the multivariable analysis performed includes all known high-risk clinical, surgical, and pathological factors in stage I lung ADC, as well as the recently described IASLC/ATS/ERS and WHO classification; (3) our study identifies a high-risk group of patients even among patients with stage IA lung ADC (*P* < 0.001); and (4) exploratory analyses included OS in addition to 5-year lung cancer–specific mortality. The fact that mPS was able to stratify prognostically different groups, even among intermediate-risk lung ADC patients, underscores the utility of this molecular signature.

Among invasive ADC, for the high-grade subtypes (MIP, SOL, and IMA), 5-year cancer-specific mortality in patients with low mPS is far lower than in patients with high mPS (11%, 8%, 0% vs 31%, 21%, and 58%). In patients with the SOL predominant subtype, who we reported have early, extrathoracic, and multisite recurrences and poor postrecurrence survival [7], 17% of patients (25/147) had low mPS, compared with 83% with high mPS. There was also strong evidence of an increasing proportion of high mPS with increasing percentage of SOL component, in all patient cohorts. These findings are plausible, as our previous study showed that mitotic counts of the SOL subtype were 2-fold greater than those of other histological subtypes, which therefore suggests that the presence and increasing percentage of SOL pattern would be strongly associated with high CCP [22]. The strong prognostic effect of CCP score, which is a quantitative and clinical laboratory independent measure of risk, supports the use of this RNA-based expression assay as an adjunct to conventional pathological features.

A major limitation of the IASLC/ATS/ERS classification is that the majority (40%–70%) of cases of stage I lung ADC are intermediate-grade ACI and PAP subtypes [3, 10, 13, 14]. In an attempt to identify a high-risk group among patients with ACI and PAP subtype tumors, we and others have reported that high mitotic counts [22], presence of the cribriform pattern [4], lack of thyroid transcription factor–1 expression [23], immunoinhibitory tumor microenvironment [8], and nuclear estrogen receptor–α expression [24] are indicative of poor prognosis. Although these pathological high-risk factors can be helpful in identifying a cohort of high-risk patients with stage I lung ADC, their qualitative and subjective aspects, interobserver variability [25, 26], and difficulty of standardization pose a practical problem when attempting to apply them universally. The biological factors underlying the described pathological information are unknown. Importantly, mPS is able to significantly distinguish the prognosis of intermediate-grade subtypes (ACI and PAP), which account for the majority of tumors (63% in our cohort) in stage I patients. Furthermore, in our study, there was no correlation between high mPS and *EGFR* or *KRAS* mutations, thereby suggesting that CCP score and mPS can provide risk information regardless of driver mutation status.

Tumor RNA signatures have shown high accuracy as prognostic markers in breast cancer [27–29]. An examination of prognostic breast RNA profiles revealed a common profile of cell cycle–regulated mRNAs [15, 27]. CCP signature has been previously shown to be a superior prognostic tool in the treatment of prostate cancer [15]. The expression levels of cell cycle genes in our study indicate that these gene profiles measure tumor growth irrespective of underlying histological grading, morphological grade, or genetic aberrations; this underscores the utility of identifying a high-risk cohort that may benefit from chemotherapy that targets cell proliferation, as well as a low-risk cohort that can forgo adjuvant therapies. Many of the CCP signature genes evaluated in our study (BIRC5, BUB1B, CDKN3, CENPF, PRC1, RRM2, and TOP2A) [30–34] have been linked to chemotherapy sensitivity.

Current NCCN guidelines provide a category 2A recommendation for the use of adjuvant therapy for patients with stage IB (T2N0R0) disease with high-risk features, which includes tumor size >4 cm [35]. In our study, when a previously identified threshold was applied [17], 27% of patients (212/799) had stage IA disease and high mPS, which is a relatively higher distribution than the originally expected rate of 15%; however, the low mPS/high mPS threshold successfully stratified 5-year cancer-specific mortality, with a nearly 4-fold difference between the two (low vs high, 4% vs 15%). In comparison, in stage IB patients with tumors >4 cm, 5-year cancer-specific survival was 100% in patients with low mPS (12% of the cohort; 6/50) compared with 68% in patients with high mPS (88%; 44/50). Conversely, in stage IB patients with tumors ≤4 cm, those with a low mPS (8%; 21/254) had 5-year cancer-specific survival of 95%, compared with 79% for those with high mPS (92%; 233/254). This suggests that many patients with stage IB disease with tumors ≤4 cm, as well as a considerable number of patients with stage IA disease, may benefit from investigation of chemotherapy, in terms of improved 5-year cancer-specific survival [36, 37].

In conclusion, our data validate an RNA expression–based prognostic signature in a large cohort of patients with stage I lung ADC. CCP score and mPS are significantly and independently associated with risk of 5-year lung cancer–specific mortality. The current NCCN criteria for evaluating the appropriateness of adjuvant chemotherapy for stage IB patients, with the possible exception of tumor size, are largely qualitative, and their measurement is subjective—thus making standardization across locations difficult, expensive, and prone to interobserver variability. This CLIA-certified quantitative PCR platform assay provides a reproducible and quantitative measure of tumor aggressiveness that can provide important prognostic information to add to conventional clinicopathological factors. The use of this measure may help advance the multidisciplinary management of stage I lung ADC by prompting investigation of chemotherapy benefit following surgical resection.

## MATERIALS AND METHODS

### Patients

A set of 1200 patients consecutively treated at Memorial Sloan Kettering Cancer Center were included in the study if they had histological stage I lung ADC as defined by the seventh edition of the AJCC/UICC TNM criteria and had undergone complete resection. Exclusion criteria were as follows: follow-up duration <30 days after lung resection, synchronous or previous cancers diagnosed within 2 years of the lung cancer resection, multiple nodules or primary lung tumors, and receipt of neoadjuvant or combination adjuvant chemotherapy and/or radiation. However, patients who received chemotherapy and/or radiation following recurrence were included. Our retrospective study was approved by the Institutional Review Board at Memorial Sloan Kettering Cancer Center (WA0269-08).

### Sample processing

From tumor blocks with areas of ≥50% tumor, 2 10-μm formalin-fixed, paraffin-embedded (FFPE) unstained slides were processed and analyzed by quantitative PCR, using a published protocol, to determine expression levels of 31 cell cycle genes and 15 housekeeping genes [15]. Passing criteria to calculate CCP score included amplification of ≥13 housekeeping genes and 22 cell cycle genes with measurable raw C_T_ values and a standard deviation of <0.5 between CCP scores from 3 replicate measurements for each sample.

### CCP score and mPS

CCP score is an unweighted average of 31 cell cycle genes normalized by the average of 15 housekeeping genes, as previously described [15]. The formula for mPS is 20 × (0.33 × CCP score + 0.52 × stage) + 15, where CCP score is rounded to the nearest tenth and stage is treated as a numerical variable (stage IA = 1; stage IB = 2). As previously published, Cox proportional hazards regression was employed with data from 3 patient cohorts to help derive the mPS formula [17]. In previous publications, a threshold for categorizing low-risk and high-risk patients was predefined as the 85th percentile of mPS; this threshold was chosen based on literature showing that approximately 15% of stage IA patients died from lung cancer within 5 years [38–42]. The threshold mPS of 27 was established to distinguish low (mPS ≤ 27) and high (mPS > 27), reflecting low- and high-risk of survival [17].

### Histological evaluation

Hematoxylin and eosin–stained tumor slides (average, 4; range, 2–10) were reviewed by two pathologists, both of whom were unaware of patient clinical outcomes. The percentage of each histological pattern was recorded in 5% increments, and, according to the IASLC/ATS/ERS and 2015 WHO classifications, tumors were classified by the predominant subtype: adenocarcinoma in situ (AIS), minimally invasive adenocarcinoma (MIA), lepidic predominant (LEP), ACI, PAP, MIP, SOL, IMA, and colloid adenocarcinoma (COL) [9, 12].

### Analysis of mutations

*EGFR* exon 19 deletion, exon 21 L858R mutation, and *KRAS* exon 2 mutation were detected as previously described [20].

### Statistical analysis

The following clinical variables were collected and used in analysis: age at diagnosis, sex (male, female), smoking status (never, former, and current), surgical procedure (pneumonectomy, bilobectomy, lobectomy, segmentectomy, and wedge resection), tumor size (centimeters, rounded to the nearest millimeter), pathological stage (IA and IB), pleural invasion (PLX/PL0, PL1, and PL2), lymphatic invasion (absent and present), vascular invasion (absent and present), and morphological grade (low = AIS, MIA, and LEP; intermediate = ACI and PAP; high = MIP, SOL, IMA, and COL).

The primary endpoint was 5-year lung cancer–specific mortality. The primary endpoint was met if the patient died within 5 years of surgery and the cause of death was lung cancer or was unknown following lung cancer recurrence. If a patient did not experience either, then the patient was censored at the date of death from other causes, if the patient died within 5 years of surgery. If the patient did not die within 5 years of surgery, then the patient was censored at the date of the last follow-up or the date 5 years after surgery, whichever came first. The exploratory endpoint was 5-year OS. The exploratory endpoint was met if the patient died of any cause within 5 years of surgery. If the patient did not die within 5 years of surgery, then the patient was censored at the date of the last follow-up or the date 5 years after surgery, whichever came first.

Linear association of CCP score with 5-year lung cancer–specific mortality, adjusted for clinical variables, was evaluated using Cox proportional hazards regression. The *P* value was based on a χ^2^ test statistic that was the difference of likelihood ratio statistics from models that excluded and included CCP score. Linear association of mPS with 5-year lung cancer–specific mortality was analyzed similarly. All reported *P* values were 2-sided. HRs with 95% CIs were reported for each interquartile range of the score distribution. The exploratory endpoint of 5-year OS was analyzed similarly. Using the log-rank test, we evaluated whether 5-year lung cancer–specific survival was significantly more favorable for patients in the low-mPS group than in the high-mPS group. The Kaplan-Meier method was used to compute survival function estimates. The relationships between high and low mPS with SOL pattern and mutation status (wild type, *EGFR*, and *KRAS*) were examined using Fisher's exact test. All analyses were performed using SAS (version 9.4; SAS Institute, Cary, NC) and *R* (version 3.1.1 or later; *R* Core Team, Vienna, Austria).
